# An ortho­rhom­bic polymorph of cerium(III) ultraphosphate, CeP_5_O_14_

**DOI:** 10.1107/S1600536808032972

**Published:** 2008-10-15

**Authors:** Jing Zhu, Wen-Dan Cheng, Hao Zhang

**Affiliations:** a Department of Material Science and Engineering, Yunnan University, Kunming, Yunnan 650091, People’s Republic of China; b State Key Laboratory of Structural Chemistry, Fujian Institute of Research on the Structure of Matter, Chinese Academy of Sciences, Fuzhou, Fujian 350002, People’s Republic of China

## Abstract

Cerium(III) ultraphosphate, CeP_5_O_14_, was synthesized by a high-temperature solution reaction between CeO_2_ and NH_4_H_2_PO_4_ in a Ce–P molar ratio of 1:12. Colourless crystals of the ortho­rhom­bic polymorph were obtained by cooling the melt of the mixture. The structure contains (P_5_O_14_)^3−^ anionic ribbons linked by distorted CeO_8_ polyhedra.

## Related literature

For applications of rare-earth ultraphosphates, see: Cole *et al.* (2000 ▶); Katrusiak & Kaczmarek (1995 ▶); Kobayashi *et al.* (1976 ▶); Schulz *et al.* (1974 ▶). For a discussion of structure types in this chemical system, see: Averbuch-Pouchot & Durif (1992 ▶). For the triclinic polymorph of CeP_5_O_14_, see: Rzaigui *et al.* (1984 ▶).

## Experimental

### 

#### Crystal data

CeP_5_O_14_
*M* *_r_* = 518.97
Orthorhombic, 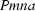

*a* = 13.1252 (12) Å
*b* = 8.7991 (9) Å
*c* = 9.0741 (9) Å
*V* = 1047.97 (18) Å^3^
*Z* = 4
Mo *K*α radiation
μ = 5.19 mm^−1^
*T* = 293 (2) K
0.08 × 0.08 × 0.05 mm

#### Data collection

Rigaku Mercury CCD diffractometer
Absorption correction: multi-scan (*CrystalClear*; Molecular Structure Corporation & Rigaku, 2001 ▶) *T*_min_ = 0.663, *T*_max_ = 0.771
7608 measured reflections
1262 independent reflections
1212 reflections with *I* > 2σ(*I*)
*R*_int_ = 0.072

#### Refinement

*R*[*F* ^2^ > 2σ(*F* ^2^)] = 0.048
*wR*(*F* ^2^) = 0.094
*S* = 1.00
1262 reflections
99 parameters
Δρ_max_ = 1.70 e Å^−3^
Δρ_min_ = −1.08 e Å^−3^

### 

Data collection: *CrystalClear* (Molecular Structure Corporation & Rigaku, 2001 ▶); cell refinement: *CrystalClear*; data reduction: *CrystalClear*; program(s) used to solve structure: *SHELXS97* (Sheldrick, 2008 ▶); program(s) used to refine structure: *SHELXL97* (Sheldrick, 2008 ▶); molecular graphics: *SHELXTL* (Sheldrick, 2008 ▶) and *DIAMOND* (Brandenburg, 2005 ▶); software used to prepare material for publication: *SHELXL97*.

## Supplementary Material

Crystal structure: contains datablocks I, global. DOI: 10.1107/S1600536808032972/bi2303sup1.cif

Structure factors: contains datablocks I. DOI: 10.1107/S1600536808032972/bi2303Isup2.hkl

Additional supplementary materials:  crystallographic information; 3D view; checkCIF report

## Figures and Tables

**Fig. 1.**
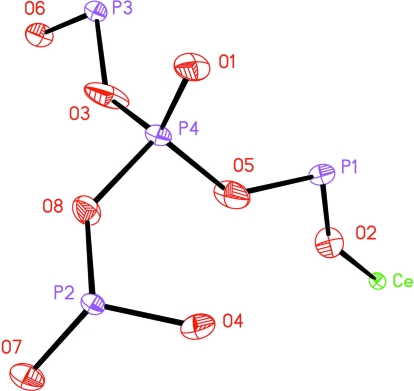
Asymmetric unit with displacement ellipsoids shown at 50% probability.

**Fig. 2.**
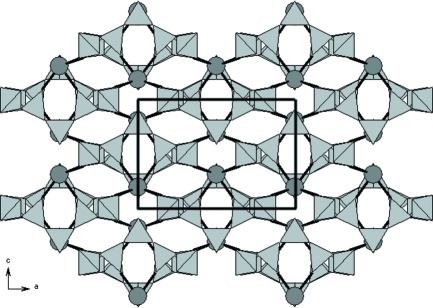
Projection of the structure along the b axis. The tetrahedra represent PO4 groups and the gray circles represent Ce3+ cations.

**Fig. 3.**
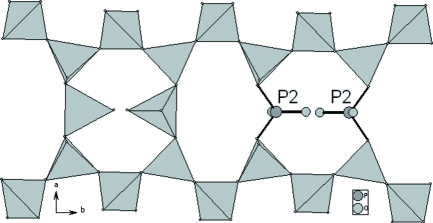
(P5O14)3- anionic ribbon running parallel to the b axis.

## supplementary crystallographic information

### Comment

Rare-earth ultraphosphates, LnP_5_O_14_ (Ln = rare-earth element), have attracted wide interest because of their potential applications in the laser domain (Schulz *et al.*, 1974; Kobayashi *et al.*, 1976; Katrusiak & Kaczmarek, 1995; Cole *et al.*, 2000). These compounds can be generally classified into four structure types: monoclinic (*P*2_1_/*a*), monoclinic (*C*2/*c*), orthorhombic (*Pnma*), and triclinic (*P*1) (Averbuch-Pouchot & Durif, 1992). In this chemical system, many of the compounds are isotypic, and some are polymorphic. However, many polymorphs of ultraphosphates LnP_5_O_14_ have not been realised to date. Herein, we present the synthesis and crystal structure of an orthorhombic polymorph of CeP_5_O_14_.

In the structure (Figs. 1 and 2), the Ce^3+^ cation plays an important bridging role, connecting neighbouring (P_5_O_14_)^3-^ anionic ribbons. The CeO_8_ polyhedron is corner-sharing with eight PO_4_ tetrahedra, with the Ce—O bond distances ranging from 2.436 (5) to 2.534 (8) Å. The shortest Ce—Ce distance is 5.2271 (9) Å. The (P_5_O_14_)^3-^ anionic ribbon may be described as two PO_4_ infinite chains linked by P(2)O_4_ tetrahedra, as shown in Fig. 3. P(1)O_4_, P(3)O_4_, and P(4)O_4_ tetrahedra are corner-shared to form screwed infinite chains along the *b* axis. P(2)O_4_ tetrahedra are corner-shared with two surrounding PO_4_ infinite chains along the *a* axis. Thus, a (P_5_O_14_)^3-^ anionic ribbon is observed parallel to *b*.

### Experimental

The title compound was prepared by a high-temperature solution reaction, using analytical reagent CeO_2_ and NH_4_H_2_PO_4_ in a molar ratio corresponding to Ce/P = 1:12. Starting mixtures were finely ground in an agate mortar to ensure optimal homogeneity and reactivity, then placed in a platinum crucible and heated at 373 K for 4 h. Afterwards, the mixtures were reground and heated to 973 K for 24 h. Finally, the temperature was cooled to 773 K at a rate of 2 K/h and air-quenched to room temperature. A few colourless, block-shaped crystals were obtained from the melt of the mixture.

### Refinement

The position of the Ce atom was obtained using direct methods, and the remaining atoms were located in succesive difference Fourier syntheses. The chemical composition of the single crystal was confirmed by energy-dispersive X-ray (EDX) analysis, and no impurity elements were detected.

### Crystal data

**Table:**

CeP_5_O_14_	*F*(000) = 980
*M**_r_* = 518.97	*D*_x_ = 3.289 Mg m^−^^3^
Orthorhombic, *P**m**n**a*	Mo *K*α radiation, λ = 0.71073 Å
Hall symbol: -P 2ac 2	Cell parameters from 2047 reflections
*a* = 13.1252 (12) Å	θ = 2.3–27.5°
*b* = 8.7991 (9) Å	µ = 5.19 mm^−^^1^
*c* = 9.0741 (9) Å	*T* = 293 K
*V* = 1047.97 (18) Å^3^	Block, colourless
*Z* = 4	0.08 × 0.08 × 0.05 mm

### Data collection

**Table:**

Rigaku Mercury CCD diffractometer	1262 independent reflections
Radiation source: fine-focus sealed tube	1212 reflections with *I* > 2σ(*I*)
graphite	*R*_int_ = 0.072
ω scans	θ_max_ = 27.5°, θ_min_ = 2.3°
Absorption correction: multi-scan (CrystalClear; Molecular Structure Corporation & Rigaku, 2001)	*h* = −17→16
*T*_min_ = 0.663, *T*_max_ = 0.771	*k* = −11→11
7608 measured reflections	*l* = −11→11

### Refinement

**Table:**

Refinement on *F*^2^	Primary atom site location: structure-invariant direct methods
Least-squares matrix: full	Secondary atom site location: difference Fourier map
*R*[*F*^2^ > 2σ(*F*^2^)] = 0.048	*w* = 1/[σ^2^(*F*_o_^2^) + 37.6801*P*] where *P* = (*F*_o_^2^ + 2*F*_c_^2^)/3
*wR*(*F*^2^) = 0.094	(Δ/σ)_max_ = 0.001
*S* = 1.00	Δρ_max_ = 1.70 e Å^−^^3^
1262 reflections	Δρ_min_ = −1.08 e Å^−^^3^
99 parameters	Extinction correction: SHELXL97 (Sheldrick, 2008), Fc^*^=kFc[1+0.001xFc^2^λ^3^/sin(2θ)]^-1/4^
0 restraints	Extinction coefficient: 0.0114 (15)

### Special details

**Table:**

Geometry. All e.s.d.'s (except the e.s.d. in the dihedral angle between two l.s. planes) are estimated using the full covariance matrix. The cell e.s.d.'s are taken into account individually in the estimation of e.s.d.'s in distances, angles and torsion angles; correlations between e.s.d.'s in cell parameters are only used when they are defined by crystal symmetry. An approximate (isotropic) treatment of cell e.s.d.'s is used for estimating e.s.d.'s involving l.s. planes.
Refinement. Refinement of *F*^2^ against ALL reflections. The weighted *R*-factor *wR* and goodness of fit *S* are based on *F*^2^, conventional *R*-factors *R* are based on *F*, with *F* set to zero for negative *F*^2^. The threshold expression of *F*^2^ > σ(*F*^2^) is used only for calculating *R*-factors(gt) *etc*. and is not relevant to the choice of reflections for refinement. *R*-factors based on *F*^2^ are statistically about twice as large as those based on *F*, and *R*- factors based on ALL data will be even larger.

### Fractional atomic coordinates and isotropic or equivalent isotropic displacement parameters (Å^2^)

**Table:**

	*x*	*y*	*z*	*U*_iso_*/*U*_eq_	
Ce	0.5000	0.72337 (7)	0.68985 (6)	0.00797 (19)	
P1	0.2936 (2)	0.5000	0.5000	0.0113 (6)	
P2	0.0000	0.3121 (3)	0.7524 (3)	0.0091 (5)	
P3	0.3233 (2)	0.0000	0.5000	0.0102 (5)	
P4	0.16332 (14)	0.2351 (2)	0.54996 (19)	0.0098 (4)	
O1	0.1129 (4)	0.2256 (7)	0.4081 (6)	0.0189 (12)	
O2	0.3474 (4)	0.5859 (7)	0.6151 (6)	0.0182 (12)	
O3	0.2451 (5)	0.1123 (8)	0.5815 (6)	0.0295 (16)	
O4	0.0000	0.4655 (9)	0.6859 (9)	0.0155 (16)	
O5	0.2151 (5)	0.3901 (7)	0.5849 (7)	0.0307 (17)	
O6	0.3778 (4)	−0.0799 (6)	0.6175 (6)	0.0165 (12)	
O7	0.0000	0.2893 (10)	0.9131 (8)	0.0176 (17)	
O8	0.0934 (4)	0.2133 (6)	0.6876 (6)	0.0161 (11)	

### Atomic displacement parameters (Å^2^)

**Table:**

	*U*^11^	*U*^22^	*U*^33^	*U*^12^	*U*^13^	*U*^23^
Ce	0.0074 (3)	0.0090 (3)	0.0075 (3)	0.000	0.000	0.0003 (2)
P1	0.0062 (11)	0.0135 (13)	0.0143 (13)	0.000	0.000	−0.0033 (11)
P2	0.0089 (12)	0.0120 (13)	0.0063 (11)	0.000	0.000	−0.0008 (10)
P3	0.0080 (11)	0.0124 (13)	0.0102 (12)	0.000	0.000	−0.0013 (10)
P4	0.0059 (8)	0.0152 (10)	0.0083 (8)	0.0002 (7)	0.0001 (6)	−0.0019 (7)
O1	0.021 (3)	0.023 (3)	0.013 (2)	0.006 (3)	−0.008 (2)	−0.001 (2)
O2	0.014 (3)	0.023 (3)	0.018 (3)	−0.006 (2)	−0.003 (2)	0.000 (2)
O3	0.029 (3)	0.050 (4)	0.010 (3)	0.028 (3)	0.001 (2)	−0.004 (3)
O4	0.019 (4)	0.012 (4)	0.016 (4)	0.000	0.000	−0.005 (3)
O5	0.034 (4)	0.031 (4)	0.027 (3)	−0.027 (3)	0.019 (3)	−0.015 (3)
O6	0.016 (3)	0.017 (3)	0.016 (3)	0.006 (2)	−0.002 (2)	0.001 (2)
O7	0.021 (4)	0.024 (4)	0.008 (3)	0.000	0.000	−0.002 (3)
O8	0.017 (3)	0.017 (3)	0.014 (2)	0.007 (2)	0.005 (2)	0.004 (2)

### Geometric parameters (Å, °)

**Table:**

Ce—O2	2.436 (5)	P2—O8^ix^	1.614 (5)
Ce—O2^i^	2.436 (5)	P2—O8	1.614 (5)
Ce—O6^ii^	2.449 (5)	P3—O6^x^	1.464 (5)
Ce—O6^iii^	2.449 (5)	P3—O6	1.464 (5)
Ce—O7^iv^	2.513 (7)	P3—O3^x^	1.606 (6)
Ce—O1^v^	2.514 (5)	P3—O3	1.606 (6)
Ce—O1^vi^	2.514 (5)	P4—O1	1.450 (5)
Ce—O4^vii^	2.534 (8)	P4—O3	1.549 (6)
P1—O2	1.470 (6)	P4—O5	1.557 (6)
P1—O2^viii^	1.470 (6)	P4—O8	1.562 (5)
P1—O5^viii^	1.609 (6)	O1—Ce^iv^	2.514 (5)
P1—O5	1.609 (6)	O4—Ce^xi^	2.534 (7)
P2—O7	1.472 (8)	O6—Ce^xii^	2.449 (5)
P2—O4	1.479 (8)	O7—Ce^vi^	2.513 (7)
			
O2—Ce—O2^i^	110.6 (3)	O2^viii^—P1—O5^viii^	106.0 (3)
O2—Ce—O6^ii^	144.55 (18)	O2—P1—O5	106.0 (3)
O2^i^—Ce—O6^ii^	74.82 (19)	O2^viii^—P1—O5	109.8 (3)
O2—Ce—O6^iii^	74.82 (19)	O5^viii^—P1—O5	100.4 (6)
O2^i^—Ce—O6^iii^	144.55 (18)	O7—P2—O4	121.9 (5)
O6^ii^—Ce—O6^iii^	81.8 (3)	O7—P2—O8^ix^	106.7 (3)
O2—Ce—O7^iv^	72.54 (17)	O4—P2—O8^ix^	110.1 (3)
O2^i^—Ce—O7^iv^	72.54 (17)	O7—P2—O8	106.7 (3)
O6^ii^—Ce—O7^iv^	76.3 (2)	O4—P2—O8	110.1 (3)
O6^iii^—Ce—O7^iv^	76.3 (2)	O8^ix^—P2—O8	98.9 (4)
O2—Ce—O1^v^	142.43 (19)	O6^x^—P3—O6	121.5 (5)
O2^i^—Ce—O1^v^	79.83 (19)	O6^x^—P3—O3^x^	105.8 (3)
O6^ii^—Ce—O1^v^	72.48 (18)	O6—P3—O3^x^	110.6 (3)
O6^iii^—Ce—O1^v^	118.08 (19)	O6^x^—P3—O3	110.6 (3)
O7^iv^—Ce—O1^v^	142.65 (14)	O6—P3—O3	105.8 (3)
O2—Ce—O1^vi^	79.83 (19)	O3^x^—P3—O3	100.5 (5)
O2^i^—Ce—O1^vi^	142.43 (19)	O1—P4—O3	116.1 (3)
O6^ii^—Ce—O1^vi^	118.08 (19)	O1—P4—O5	115.5 (4)
O6^iii^—Ce—O1^vi^	72.48 (18)	O3—P4—O5	105.7 (4)
O7^iv^—Ce—O1^vi^	142.65 (14)	O1—P4—O8	115.8 (3)
O1^v^—Ce—O1^vi^	72.2 (3)	O3—P4—O8	100.0 (3)
O2—Ce—O4^vii^	71.29 (16)	O5—P4—O8	101.6 (3)
O2^i^—Ce—O4^vii^	71.29 (16)	P4—O1—Ce^iv^	163.4 (4)
O6^ii^—Ce—O4^vii^	138.78 (13)	P1—O2—Ce	148.0 (4)
O6^iii^—Ce—O4^vii^	138.78 (14)	P4—O3—P3	141.9 (4)
O7^iv^—Ce—O4^vii^	113.9 (3)	P2—O4—Ce^xi^	129.5 (5)
O1^v^—Ce—O4^vii^	79.0 (2)	P4—O5—P1	135.1 (4)
O1^vi^—Ce—O4^vii^	79.0 (2)	P3—O6—Ce^xii^	148.7 (3)
O2—P1—O2^viii^	122.6 (5)	P2—O7—Ce^vi^	174.7 (6)
O2—P1—O5^viii^	109.8 (3)	P4—O8—P2	132.2 (4)

Symmetry codes: (i) −*x*+1, *y*, *z*; (ii) −*x*+1, *y*+1, *z*; (iii) *x*, *y*+1, *z*; (iv) −*x*+1/2, −*y*+1, *z*−1/2; (v) *x*+1/2, −*y*+1, *z*+1/2; (vi) −*x*+1/2, −*y*+1, *z*+1/2; (vii) *x*+1/2, *y*, −*z*+3/2; (viii) *x*, −*y*+1, −*z*+1; (ix) −*x*, *y*, *z*; (x) *x*, −*y*, −*z*+1; (xi) *x*−1/2, *y*, −*z*+3/2; (xii) *x*, *y*−1, *z*.
